# Risk factors of central catheter bloodstream infections in intensive care units: A systematic review and meta-analysis

**DOI:** 10.1371/journal.pone.0296723

**Published:** 2024-04-23

**Authors:** Huayong Huang, Qiaoling Chang, Yanhui Zhou, Li Liao

**Affiliations:** 1 The First Affiliated Hospital of South China University, Hengyang, Hunan, China; 2 University of South China, Hengyang, Hunan, China; AIIMS: All India Institute of Medical Sciences, INDIA

## Abstract

**Background:**

Central catheter bloodstream infections (CRBSI) is a major cause of healthcare-associated infections. However, few factors are generally accepted and some studies have conflicting finding about some factors, possibly caused by limitation associated with an individual study. This study was to identify risk factors for CRBSI in intensive care units.

**Methods:**

We searched the PubMed, Cochrane Library, Web of science and EMBASE databases and the 4 top Chinese-language databases, including WanFang data, China National Knowledge Infrastructure (CNKI), and Chinese Science and Technology Journal Database (VIP), China Biology Medicine disc (CBM) as of July 2023. Case control and cohort studies were included. Two authors independently screened the literature and evaluated the quality of the studies using the Newcastle-Ottawa scale (NOS). The pooled effect size was estimated using the odds ratio (OR), and the corresponding 95% confidence interval (CI) was calculated. The Cochrane Q (χ2) and I2 tests were used to assess heterogeneity among studies, and each risk factor was tested for its robustness using fixed- or random-effects models.

**Findings:**

A total of 32 studies enrolled, among which eleven factors were identified, they were divided into two categories: modifiable and unmodifiable factors. Modifiable factors: duration of catheterization (≥ 5d) (OR: 2.07, 95%CI: 1.41–3.03), duration of catheterization (≥ 7d) (OR: 3.62, 95%CI: 2.65–4.97), duration of catheterization (≥ 14d)(OR: 4.85, 95%CI: 3.35–7.01), total parenteral nutrition (OR: 2.27,95%CI: 1.56–3.29), use of multiple-lumen catheters(OR: 3.41, 95%CI: 2.27–5.11), times of tube indwelling (OR: 3.50, 95%CI: 2.93–4.17), length of ICU stay (OR: 4.05, 95%CI: 2.41–6.80), the position of indwelling(OR: 2.41, 95%CI: 2.03–2.85); Unmodifiable factors: APACHEII scores (OR: 1.84, 95%CI: 1.54–2.20), Age≥ 60 years old (OR: 2.19, 95%CI: 1.76–2.73), the extensive use of antibiotic (OR: 3.54, 95%CI: 1.65–7.61), Diabetes mellitus (OR: 3.06, 95%CI: 2.56–3.66), Immunosuppression (OR: 2.87, 95%CI: 2.08–3.95).

**Conclusions:**

Effective interventions targeting the above modifiable factors may reduce the risk of developing CRBSI in ICU and improve the clinical outcome of patients. Further prospective studies are needed to confirm these findings.

## Introduction

Catheter-related bloodstream infection (CRBSI) is defined as the presence of bacteremia originating from an intravenous catheter [1]. CRBSI is a prevalent and significant problem in the adult ICU, with an estimated 4.59 per 1,000 catheter days (95% CI: 2.31–6.86) each year, with the highest rate in non-tunneled central venous access devices (CVADs) [2]. And CRBSI is associated with significant morbidity and mortality, health care costs and lengths of stay [3, 4]. CRBSI is a major cause of healthcare-associated infections [5], and were found to be the most costly HAIs at $45,814 (95% CI, $30,919-$65,245) [6]. In practice, as many as 65%-70% of cases of CRBSI can be prevent [7]. Some risk factors have been reported, and some of them are strongly associated with the occurrence of CRBSI, included modifiable and unmodifiable factors [8], such as duration of catheterization, the position of indwelling and so on. However, few factors are generally accepted and some studies have conflicting finding about some factors, possibly caused by limitation associated with an individual study. Besides, CRBSI reduce the benefit of the catheterization. Thus, the meta-analysis about CRBSI is necessary.

A previous meta-analysis in 2018 show that risk factors for CRBSI in ICU patients included duration of indwelling catheter, multiple-lumen catheters, femoral vein catheterization and so on [9]. However, more studies focused on CRBSI in ICU patients have appear in last five years. We also find a meta-analysis [10] in 2022 update the risk factors of CRBSI in ICU patients in China, while the risk factors of duration of catheterization in this study weren’t perform subgroup analysis, and some risk factors about length of ICU stay, the extensive use of antibiotics and immunosuppression are not analyzed. In addition, that meta-analysis didn’t screen EMBASE databases and China Biology Medicine disc (CBM). We hope to perfect the meta-analysis. Therefore, we performed a meta-analysis to identified the risk factors of CRBSI in ICU.

## Materials and methods

Our protocol was prospectively registered in PROSPERO (CRD42021262052), an international prospective register database of systematic review protocol on health-related topic.

### Publication search

For this analysis, we searched the PubMed, Cochrane Library, Web of science and EMBASE databases and the 4 top Chinese- language databases, including Wan Fang data, China National Knowledge Infrastructure (CNKI), and Chinese Science and Technology Journal Database (VIP), China Biology Medicine disc (CBM) as of July 2023 (cut-off date 16st July 2023). The following Medical Subject Heading (MESH) terms in PubMed database were used: “(Intensive Care Units OR Intensive Care Unit OR Unit, Intensive Care OR Unit, Intensive Care) AND(Central Venous Catheters OR Catheter, Central Venous OR Catheters, Central Venous OR Venous Catheter, Central OR Venous Catheters, Central OR Central Venous Catheter) OR (Catheterization, Central Venous OR Venous Catheterization, Central OR Central Catheterization OR Catheterization, Central OR Catheterizations, Central OR Central Catheterizations OR Central Venous Catheterization OR Catheterizations, Central Venous OR Central Venous Catheterizations OR Venous Catheterizations, Central) AND infections. Additional studies were assessed by screening the reference lists of relevant studies. The detailed search strategy for each database is provided in the S1 File.

### Inclusion and exclusion criteria

The PICOS categories (i.e., population, intervention, comparator, outcomes, and study design) were used to define study inclusion criteria. The following inclusion criteria of the meta-analysis were used:(1) patients having traditional central venous catheter(CVC) under key, neck veins, femoral venous tubes, not including Peripherally Inserted Central Venous Catheters(PICC), arterial cannulation; (2) age ≥ 18 years old and not including hematological malignant tumor; (3) estimation of the association between any risk factors and the central venous catheter bloodstream infections (CVC-BSI) or central line–associated bloodstream infection (CLABSI); (4) outcome measure regarding CVC-BSI or CLABSI is a standardized definition and reported by the dedicated personnel; (5) reported relative risk (RR) or odds ratio (OR) with corresponding 95% confidence interval (CI) for CVC-BSI or CLABSI; (6) case-controlled or cohort studies. Review studies, conference abstracts, study protocols, letters, animal studies, and studies without adequate data and overlapping data were excluded from the analysis.

### Data extraction and quality assessment

Data from the included studies were extracted and summarized independently by two of the authors. Any disagreement was resolved by the adjudicating senior authors. The following data was extracted for each study: first author, publication year, recruitment period, study design, country, risk factors, CRBSI definition, CRBSI diagnostic, cases/controls (case-controlled studies), and the score of Newcastle Ottawa scale (NOS). The quality assessment was evaluated by NOS [11]. We preferred multivariate risk ratios (RRs)/odd ratios (ORs) with corresponding 95% confidence intervals (CIs) rather than univariate results. Methodological quality was evaluated the recruitment of cases and controls, the between-study comparability, and the ascertainment of the outcome or the exposure (the more the stars, the higher of the studies; studies with ≥6 stars were considered to be high-level quality, and the related results are shown in Table 1). We used the Meta-analysis Of Observational Studies in Epidemiology (MOOSE) reporting guidelines [12]. The final results are provided in the S1 and S2 Tables.

**Table 1:** General characteristics of the included studies in the meta-analysis.

Study year	Nation	Numbers of patients	Study type	Recruited period	CRBSI diagnosis	Risk factors
Hong Zhou [13] 2012	China	4516	Cohort study	2010.7–2011.6	blood culture	2.3.4
Huiying Yang [14] 2018	China	220	Case-control	2015. 1–2017.12	ISDA	2.3.5.6.7.8
Li Xiao [15] 2012	China	174	Cohort study	2007.6–2008.5	CDC	9.10
Yinmei Liu [16] 2014	China	1677	Cohort study	2008. 1–2012.12	blood culture	3.5.6
Xinqun Pan [17] 2018	China	522	Case-control	2016. 1–2017.12	Catheter cultures and blood culture	3.6.7.13
Shizhi Tian [18] 2018	China	381	Case-control	2012. 1–2016.12	CDC	3. 11.16
Baochun Zhou [19] 2015	China	245	Cohort study	2011.1–2013.12	CMA	3.7.8. 11.17
Zhihuang Jiang [20] 2016	China	1330	Case-control	2012. 1–2014.12	CDC	3.8.18
Bing Liu [21] 2015	China	103	Case-control	2014.9–2014.12	Catheter cultures and blood culture	3.12
Xijiang Zhang [22] 2012	China	623	Case-control	2008. 1–2011.8	Catheter cultures and blood culture	3.5.7.8.12.16.19
Yuanye Li [23] 2020	China	2445	Case-control	2014–2018	CDC	3.20.21.22
Hongying Chen [24] 2020	China	398	Case-control	2018. 1–2019.12	Catheter cultures and blood culture	3.6.7. 11.14.17
Dongmei Hou [25] 2021	China	256	Case-control	2017.8–2019.3	CDC	3.5.8. 11.16.23
Huifen Wang [26] 2012	China	249	Case-control	2010. 1–2011.8	CMA	3.7.16
Liyan Chen [27] 2015	China	200	Case-control	2013. 1–2014.5	Catheter cultures	3.14.22
Zhen Tao [28] 2017	China	483	Case-control	2010.4–2014.4	Catheter cultures and blood culture	3.23
Yanfang Yang [29] 2017	China	695	Case-control	2014.10–2016. 12	CMA	6.7. 11.22.19
Ye Liang [30] 2018	China	252	Case-control	2016.4–2017.4	CMA	3.14.30
Yiyue Zhong [31] 2021	China	686	Cohort study	2009–2018	IDSA	14.20.31.32.33.34
S.B. MISHRA [32] 2017	India	153	Cohort study	2014. 1–2015.4	blood culture	3.17
Zied Hajjej [33] 2014	Tunis	260	Case-control	2012. 1–2012.8	IDSA	3.14.15.30
Peng, S [34] 2013	China	174	Cohort study	2007.6–2008.5	CDC	1.35
Matthew E. Lissauer [35] 2012	American	961	Cohort study	2008.4–2009.6	CDC	36.37.38.39.40.41
Daniela Bicudo [36] 2011	Brazil	555	Cohort study	2005. 11–2006.10	CDC	3.12
Bo Yang [37] 2016	China	358	Case-control	2013.2–2015. 1	CDC	3.5.8. 11.14.20
Li Liu [38] 2022	China	739	Case-control	2018. 1–2020. 12	Catheter cultures	3.8.9. 11.14
Kaichen Yan [39] 2022	China	252	Case-control	2020. 1–2021. 1	Catheter cultures	3. 11.16
HairongYuan [40] 2021	China	765	Case-control	2018.3–2020.9	IDSA	3.7. 11.24.42.43
Li, Qiao [41] 2020	China	674	Case-control	2016. 1–2018. 12	IDSA	3.23
Huaming Peng [42] 2020	China	313	Case-control	2018. 1–2019. 12	IDSA	3.8.14.23
Xiaoqing Shao [43] 2018	China	1276	Cohort study	2014.7–2015.6	CDC	3.8.9.24.44
Shuiqin Cheng [44] 2016	China	171	Case-control	2010.4–2015.5	Catheter cultures and blood culture	3.7.8.17

1.the applications of multiple antibiotics before CRBSI 2. the placement of indwelling3.Duration of catheterization 4.the categories of ICU 5. times of tube indwelling 6. length of ICU stay 7.APACHEII scores 8. The position of indwelling 9. Application one or more antibiotics 10. multiple central lines 11.age 12.use of multiple-lumen catheters 13.application broad-spectrum antibiotics≥14d 14. Diabetes mellitus 15. coexistent infections 16. total parenteral nutrition 17. Immunosuppression 18. Traditional locating method by anatomical way of hemodialysis catheter 19. urgent intubations 20. Temperature 21. hemoglobin 22. Serum albumin content 23.the extensive use of antibiotics 24. basic disease 30. One or more antibiotics before insertion 31. Acute Respiratory Distress Syndrome 32. arterial hypotension (MAP<70 mmHg) 33. Creatinine increase (≥133lmol/L) 34. continuous renal replacement therapy,35. multiple central lines 36. Male 37.ICU transfer for higher level of care 38.Emergency surgery 39.Charlson comorbidity index (≥2) 40.National predicted ICU mortality Quartile 2 41. reopening of recent laparotomy 42. mechanical ventilation 43. the number of broad-spectrum antibiotics 44. catheter type.

### Statistical analyses

The heterogeneity was estimated using the Cochran’s Q (χ2) test and the inconsistency (I2) statistic. If I2 < 50% and (or) p ≥ 0. 1 we would use a fixed effect model. And if I2 ≥ 50% and (or) P <0. 1, the heterogeneity is large, the random effect model is adopted. Furthermore, a sensitivity analysis was done to identify potential model. Review Manager software (version 5.3; The Nordic Cochrane Centre, Copenhagen, Denmark) was used for statistical analysis, a p value less than 0.05 was considered statistically significant.

## Results

### Study characteristics

**Fig 1:**
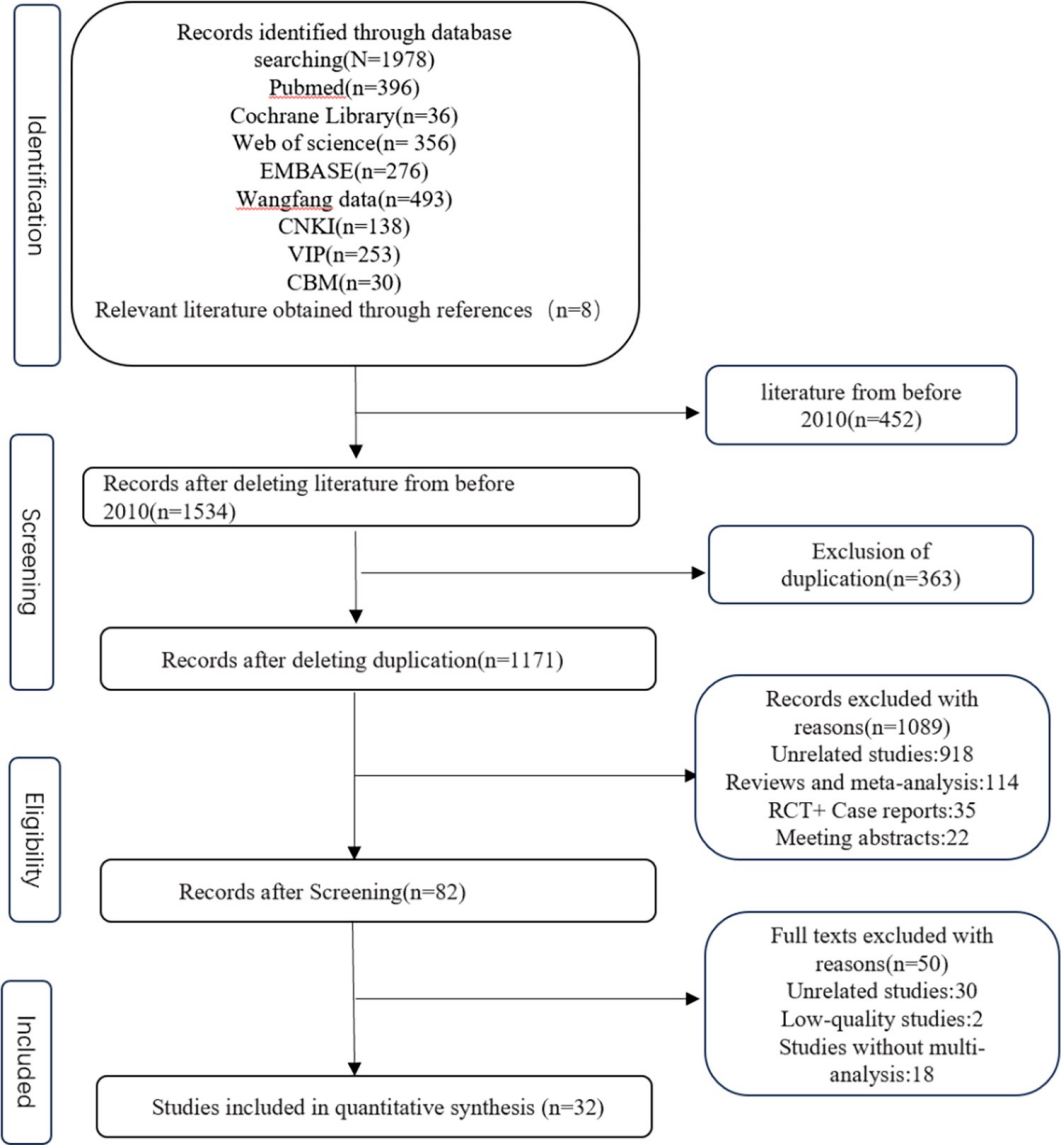
Flow chart of the review process.

This search initially provided 1986 hits, of which 1171 were screened by two independent investigators. After screening topics and abstracts, 1089 studies were excluded. After a full-text review, 32 studies were finally included. All studies identified patients who have CRBSI in ICU and investigated the risk factors of CRBSI. The flow chart of the literature screening is shown in Fig 1. The baseline characteristics of the included studies are presented in Table 1.

Initially, we found 44 risk factors from the 32 studies. Of these, 32 factors could not be quantitatively analyzed in the present study due to insufficient data. In the succeeding meta-analysis, the factor of Serum albumin content cannot analyze due to heterogeneity. Thus, reported in at least three studies, 11 risk factors remained.

### Duration of catheterization

**Fig 2:**
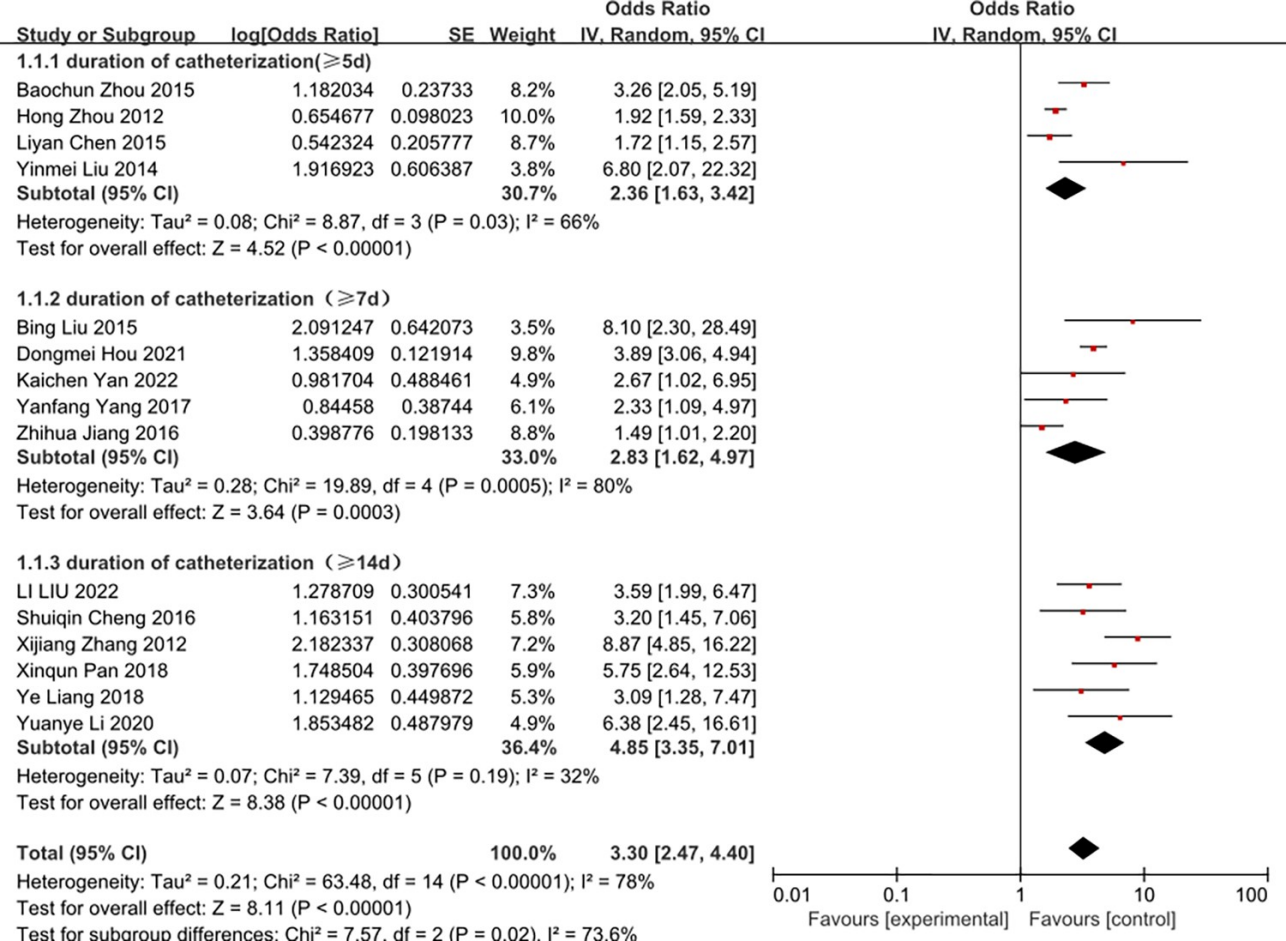
The forest plot shows the relationship between duration of catheterization and the risk of CRBSI.

**Fig 3:**
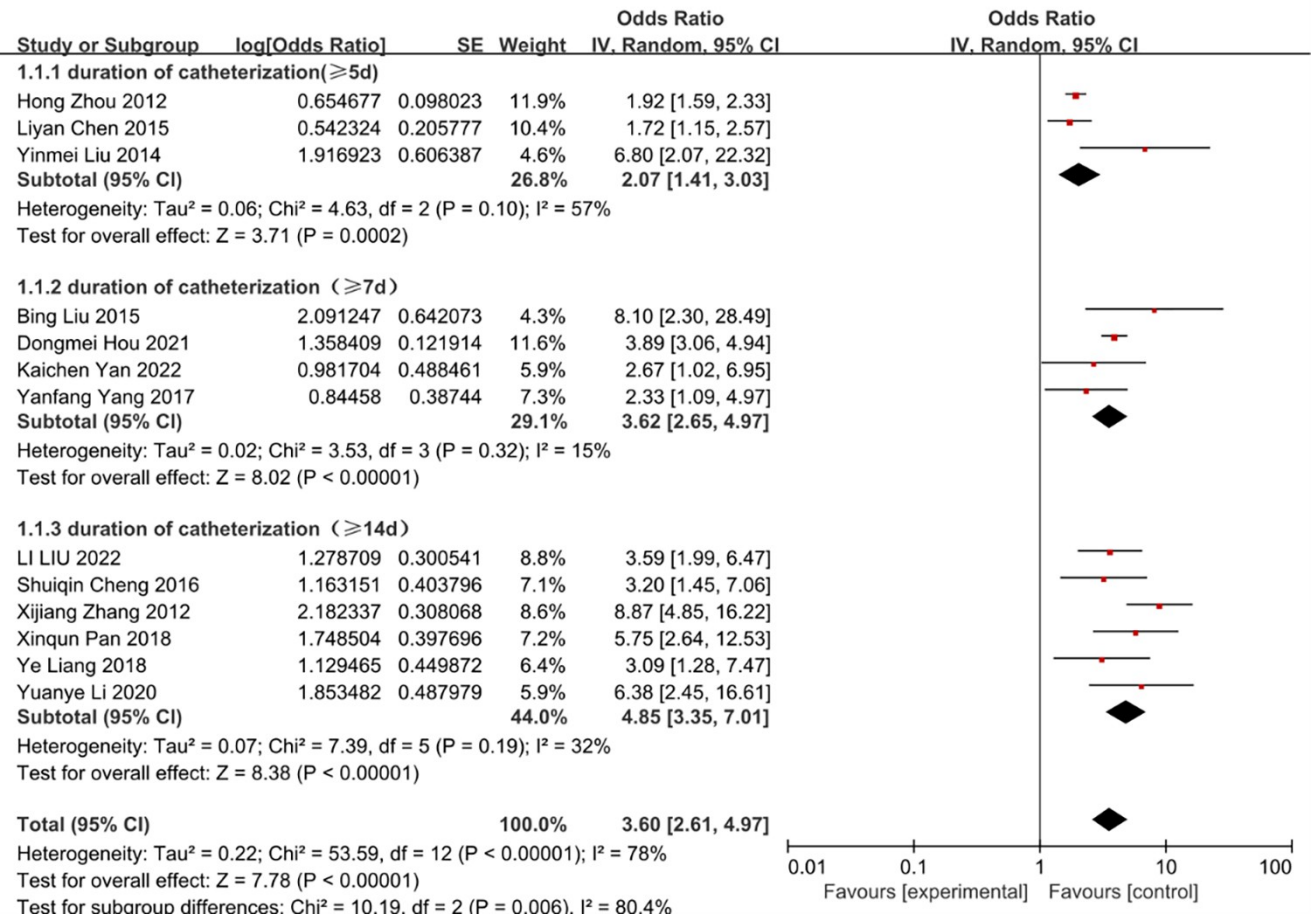
The forest plot shows the relationship between duration of catheterization and the risk of CRBSI (adjusting).

A total of 15 studies investigated the impact of duration of catheterization in CRBSI. Most of them identified duration of catheterization as a significant predictive factor. The heterogeneity of duration of catheterization (≥5d) was reduced from 66 to 57% by removal of the study of zhou 2015 et al. The heterogeneity of duration of catheterization (≥7d) was reduced from 80% to 15% by removal of the study of Jiang 2016 et al. (Figs 2 and 3).

### Times of tube indwelling

**Fig 4:**
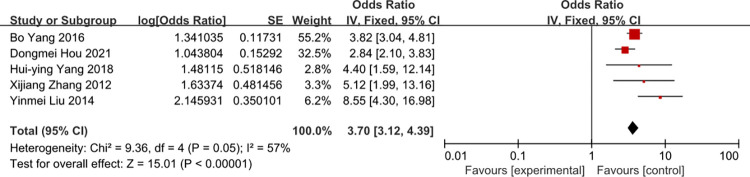
The forest plot shows the relationship between times of tube indwelling and the risk of CRBSI.

**Fig 5:**
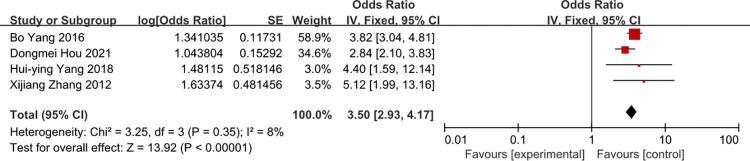
The forest plot shows the relationship between times of tube indwelling and the risk of CRBSI (adjusting).

Five studies were included in the meta-analysis. The heterogeneity of times of tube indwelling was reduced from 57 to 8% by removal of the study of Liu 2014. There was a strong likelihood of a positive liner relationship between times of tube indwelling and the rate of CRBSI (OR = 3.50, 95%CI:2.93–4.17) (Figs 4 and 5).

### Length of ICU stay

**Fig 6:**
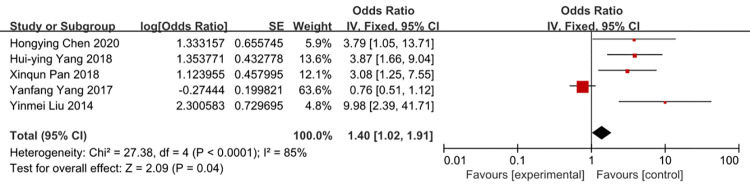
The forest plot shows the relationship between length of ICU stay and the risk of CRBSI.

**Fig 7:**
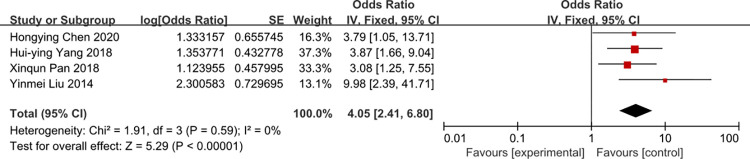
The forest plot shows the relationship between length of ICU stay and the risk of CRBSI (adjusting).

A meta-analysis of five studies that reported on demonstrated time of ICU stay was associated with the risk of CRBSI (OR = 4.05, 95%CI: 2.41–6.80, I2 = 0%) (Figs 6 and 7). The heterogeneity of times of tube indwelling was reduced from 85 to 0% by removal of the study of Yang 2017 et al.

### APACHEII scores

**Fig 8:**
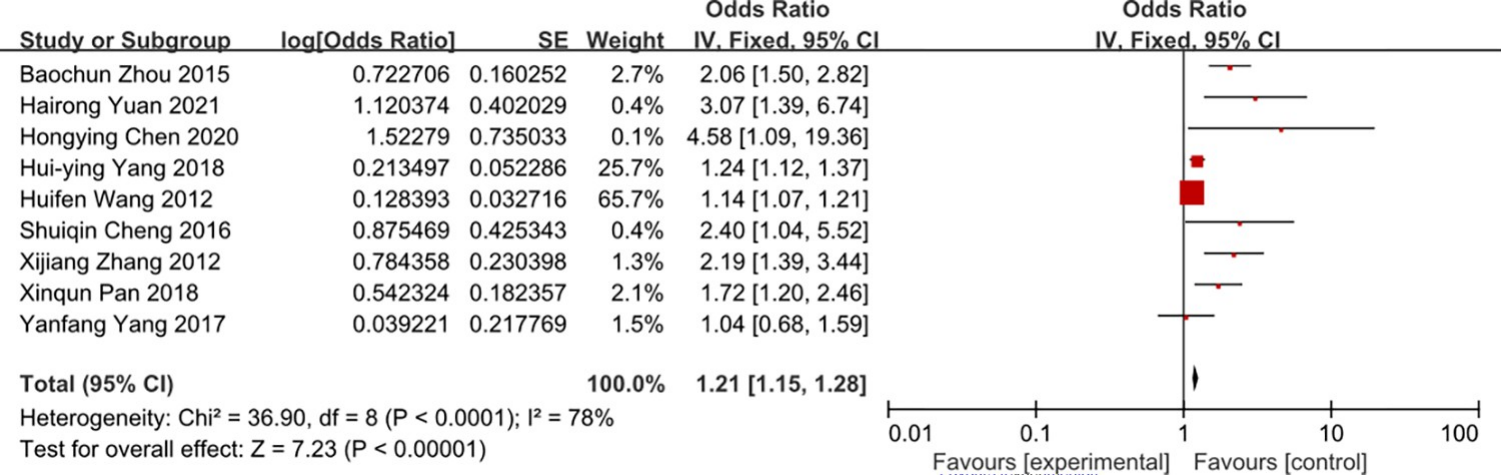
The forest plot shows the relationship between APACHEII scores and the risk of CRBSI.

**Fig 9:**
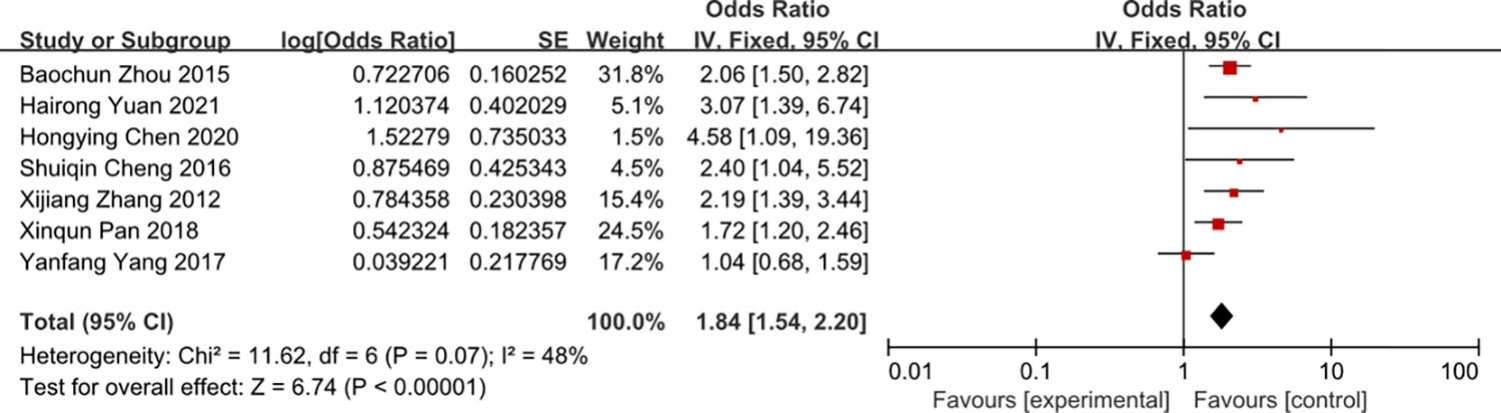
The forest plot shows the relationship between APACHEII scores and the risk of CRBSI (adjusting).

A meta-analysis of four studies APACHEII scores was associated with the risk of CRBSI (OR = 1.84, 95%CI: 1.54–2.20, I2 = 48%) (Figs 8 and 9). The heterogeneity of times of tube indwelling was reduced from 78% to 48% by removal of the study of Yang 2018 and Wang 2012 et al.

### The position of indwelling

**Fig 10:**
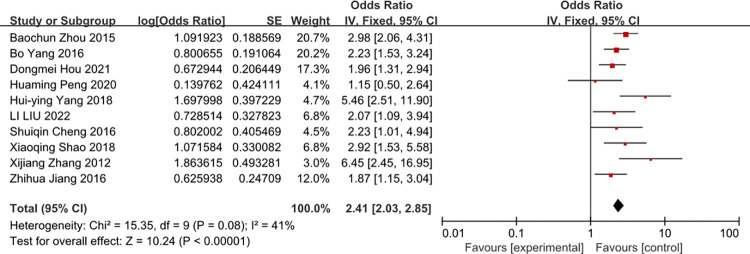
The forest plot shows the relationship between the position of indwelling and the risk of CRBSI Age ≥ 60 years old.

A meta-analysis often studies of the position of indwelling was associated with the risk of CRBSI (OR = 2.41, 95%CI: 2.03–2.85, I2 = 41%) (Fig 10).

**Fig 11:**
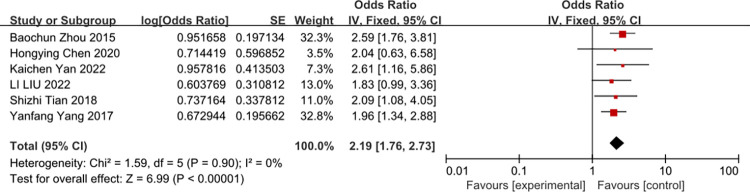
The forest plot shows the relationship between Age≥60 years old and the risk of CRBSI.

A meta-analysis of six studies Age≥60 was associated with the risk of CRBSI (OR = 2.19, 95%CI: 1.76–2.73, I^2^ = 0%) (Fig 11).

### The extensive use of antibiotics

**Fig 12:**
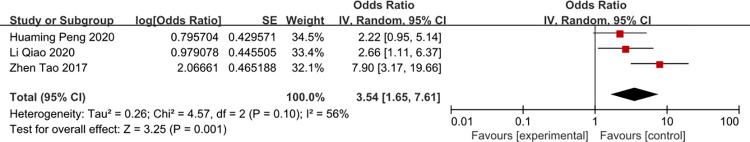
The forest plot shows the relationship between the extensive use of antibiotics and the risk of CRBSI.

A meta-analysis of three studies the extensive use of antibiotics was associated with the risk of CRBSI (OR = 3.54, 95%CI: 1.65–7.61, I 2 = 56%) (Fig 12).

### Use of multiple-lumen catheters

**Fig 13:**
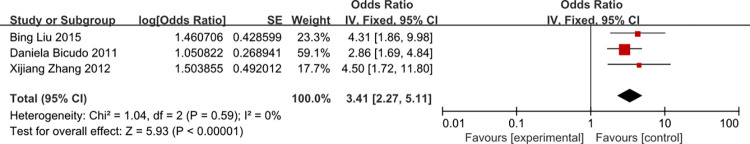
The forest plot shows the relationship between use of multiple-lumen catheters and the risk of CRBSI.

A meta-analysis of three studies use of multiple-lumen catheters was associated with the risk of CRBSI (OR = 3.41, 95%CI: 2.27–5.11, I 2 = 0%) (Fig 13).

### Diabetes mellitus

**Fig 14:**
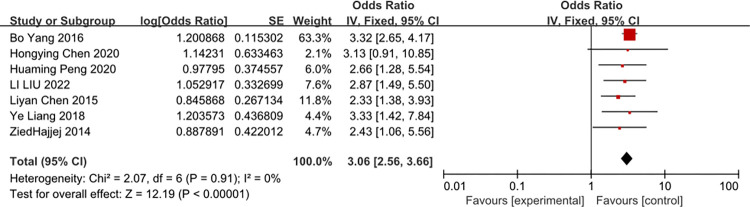
The forest plot shows the relationship between Diabetes mellitus and the risk of CRBSI.

A meta-analysis of three studies Diabetes mellitus was associated with the risk of CRBSI (OR = 3.06, 95%CI: 2.56–3.66, I 2 = 0%) (Fig 14).

### Total parenteral nutrition

**Fig 15:**
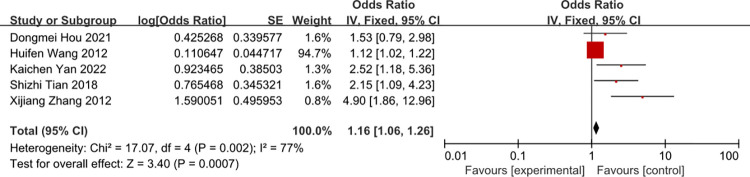
The forest plot shows the relationship between total parenteral nutrition and the risk of CRBSI.

**Fig 16:**
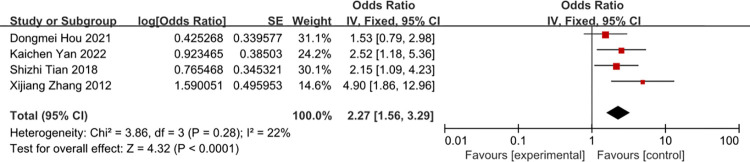
The forest plot shows the relationship between total parenteral nutrition and the risk of CRBSI (adjusting).

A meta-analysis of five studies total parenteral nutrition was associated with the risk of CRBSI (OR = 2.27, 95%CI: 1.56–3.29 (Figs 15 and 16). The heterogeneity of times of tube indwelling was reduced from 77% to 22% by removal of the study of Wang 2012.

### Immunosuppression

**Fig 17:**
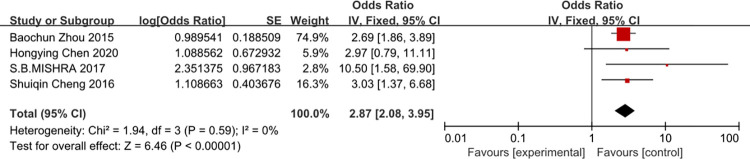
The forest plot shows the relationship between immunosuppression and the risk of CRBSI.

A meta-analysis of four studies immunosuppression was associated with the risk of CRBSI (OR = 2.87, 95%CI: 2.08–3.95, I^2^ = 0%) (Fig 17).

## Discussion

In this study, meta-analysis found that duration of catheterization, times of tube indwelling, use of multiple-lumen catheters, length of ICU stays, the position of indwelling, age>60, the extensive use of antibiotics, APACHEII scores, diabetes mellitus, total parenteral nutrition and immunosuppression were significant risk factors for CRBSI. The risk factors of CRBSI include two categories: modifiable and unmodifiable factors [8]. We could early to intervene modifiable factors to prevent the occurrence of CRBSI.

As expected, our study confirms the view that duration of catheterization is a modifiable factor increasing the incidence of CRBSI. The meta-analysis has proved that duration of catheterization (≥5d), duration of catheterization (≥7d) and duration of catheterization (≥14d) have 2.07, 3.62 and 4.85 folds for developing CRBSI, respectively. We may conclude that the longer of the duration of catheterization, the chance of infection will increase. We advise clinician to make a check-list to evaluate the patient, if the patient didn’t need the central venous catheter, the central venous catheter can be removed to avoid the CRBSI.

Our pooled results showed that patients with tube indwelling more than one time have a 3.50-fold risk of getting CRBSI compared to patients with tube indwelling one time. On the one thing, some studies [45] consider that if the tube indwelling is difficult, the same number of pastes is over 2 times, the opportunity of the infection would be increases. On another things, blindness, repeated puncture route could be largely damage to local subcutaneous tissue and intravascular inner wall, to increase the possibility of inflammatory reactions and catheter infections, which could lead to CRBSI. Tube indwelling with ultrasonic is popular in intensive care unit, ultrasonic can reduce the times of tube indwelling. Thus, it can prevent CRBSI. So medical work needs to improve their skills and minimize the times of tube indwelling.

A meta-analysis show that the femoral vein compared with the internal jugular vein or the subclavian vein are likely to develop CRBSI. If you only to reduce CRBSI, the subclavian vein is more suitable position to choose [46]. A meta-analysis [47] by Cameron showed that multiple-lumen catheters were common when compared with single-lumen catheter (OR = 2.15). And this meta wasn’t correct other risk factors. And the advantage of our study corrected other risk factors, showed that patients use of multiple-lumen catheters has a 3.41-fold risk of getting CRBSI compared to patient’s use of single-lumen catheter. Templeton A et al. [48] have also revealed that number of lumens was the independent risk factors for CRBSI. If it is used for intravenous infusion, single-lumen catheter may be the best; If it is used for continuous renal replacement therapy, multiple-lumen catheters may be convenience.

At the same time, unmodifiable factors included age, APACHEII scores, diabetes mellitus. The present study showed that age of at least 60 years old was an independent risk factor of CRBSI. The majority of study enrolled in this meta were in China, and therefore the 161 results of age of at least 60 of the study might be applicable in China, and more study to conduct in other country. Thus, we concluded that age of at least 60 was an independent risk factor of CRBSI.

For APAHEII scores variable, in the study, APACHEII scores was associated with the risk of CRBSI, patients with more APACHEII scores, and it’s more likely to develop CRBSI. No sufficient evidence showed the critical value of APACHEII scores, so it needs some perspective study to confirm the critical value. Surprisingly, a study [49] showed that the APACHE II on admission is not a useful predictor for surveillance of nosocomial infection in ICUs. We considered that some risk factors influence the APACHEII scores, the patient with more APACHEII scores will receive more invasive treatment and it may increase the incidence of CRBSI, so we advices medical works to monitor the patient with more APACHEII scores.

Remarkably, our study showed that diabetes mellitus is the independent predictor of CRBSI. But Yiyue Zhong study [31] showed that a relatively higher risk of CRBSIs in patients with the glucose level of >10 mmol/L. Hyperglycemia is a common complication of critical illness [50]. So we consider that diabetes mellitus with the glucose level of >10 mmol /L is associated with CRBSI. And it needs some study to confirm this view.

To the best of our knowledge, this is the first meta-analysis to obtain a powerful conclusion between the extensive use of antibiotics and the risk of CRBSI. The extensive use of antibiotics is an independent risk of CRBSI. We think that patients with the extensive use of antibiotics, the physical condition is more badly, the ability of anti-infection became more worse, they are more likely to develop CRBSI.

Exposition to TPN increases the risk to CRBSI in adult patients with CVC and this risk raise with exposition time [51]. Not surprisingly, our study showed that immunosuppression was an independent risk factor of SSI (OR = 2.87). The reasons may be explained by the patients with immunosuppression, they more were likely to infected.

The other known risk factors have been previously reported in many studies, such as coexistent infections and application one or more antibiotics, and so on. And some risk factors such as poor hand hygiene of operator and sliding /slipping out of catheter, the medical work should be carried out correctly routinely.

## Limitation

The other known risk factors have been previously reported in many studies cannot analyzed due to the number of articles less than three, we hope to further research in the future; In the search, only included English and Chines, may cause the omission of certain factors, making the influencing factors of primary osteoporosis cannot be summarized.

## Conclusion

Effective interventions targeting the above modifiable factors may reduce the risk of developing CRBSI in ICU and improve the clinical outcome of patients. Further prospective studies are needed to confirm these findings.

## Supporting information

S1 Checklist PRISMA 2020 checklist. (DOCX)

S1 File Detailed search strategies for each database. (PDF)

S1 Table Study quality assessment of included case-control studies using NOS. (PDF)

S2 Table Study quality assessment of included cohort studies using NOS. (PDF)

S1 Data (XLSX)
