# Supplementary material for: Risk factors of central catheter bloodstream infections in intensive care units: A systematic review and meta-analysis
Source: PLoS One. 2024 Apr 23;19(4):e0296723. doi: 10.1371/journal.pone.0296723 (PMC11037535; doi:10.1371/journal.pone.0296723)
Supplement: S1 File — (PDF) [file pone.0296723.s002.pdf]

S1 file. Detailed search strategies for each database

PubMed:

((((((("Central Venous Catheters"[Mesh]) OR (((((Catheter, Central Venous[Title/Abstract]) OR (Catheters, Central Venous[Title/Abstract])) OR (Venous Catheter, Central[Title/Abstract])) OR (Venous Catheters, Central[Title/Abstract])) OR (Central Venous Catheter[Title/Abstract]))) OR ("Catheterization, Central Venous"[Mesh])) OR (((((((((Venous Catheterization, Central[Title/Abstract]) OR (Central Catheterization[Title/Abstract])) OR (Catheterization, Central[Title/Abstract])) OR (Catheterizations, Central[Title/Abstract])) OR (Central Catheterizations[Title/Abstract])) OR (Central Venous Catheterization[Title/Abstract])) OR (Catheterizations, Central Venous[Title/Abstract])) OR (Central Venous Catheterizations[Title/Abstract])) OR (Venous Catheterizations, Central[Title/Abstract]))) AND ("Infections"[Mesh])) OR ((Catheter Related Blood Stream Infection[Title/Abstract]) OR (central line-associated bloodstream infection[Title/Abstract]))) AND (("Intensive Care Units"[Mesh]) OR (((Intensive Care Unit[Title/Abstract]) OR (Unit, Intensive Care[Title/Abstract])) OR (ICU Intensive Care Units[Title/Abstract])))) AND ((relative[Title/Abstract] AND risk*[Title/Abstract]) OR (relative risk[Text Word]) OR risks[Text Word] OR cohort studies[MeSH:noexp] OR (cohort[Title/Abstract] AND stud*[Title/Abstract]))

CBM:

(("相关因素"[常用字段:智能]) OR ("高危因素"[常用字段:智能]) OR ("影响因素"[常用字段:智能]) OR ("危险因素"[不加权:扩展])) AND ((("重症监护室患者"[常用字段:智能]) OR ("ICU 患者"[常用字段:智能])) AND (("导管相关血流感染"[常用字段:智能]) OR ("中央导管相关血流感染"[常用字段:智能]) OR (("感染"[不加权:扩展]) AND ("中心静脉导管"[不加权:扩展]))))

VIP:

(((((摘要=中心静脉导管 AND 摘要=感染) OR 摘要=中央导管相关血流感染) OR 摘要=导管相关血流感染) AND (摘要=ICU患者 OR 摘要=重症监护室患者)) AND (((摘要=高危因素 OR 摘要=相关因素) OR 摘要=危险因素) OR 摘要=影响因素))

Web of science

1: TS=(Catheter, Central Venous OR Catheters, Central Venous OR Venous Catheter, Central OR Venous Catheters, Central OR Central Venous Catheter OR Central Venous Catheterss) •ĐˆLeåg : Sun Jul 16 2023 18:23:12 GMT+080[illegible]

2: TS=(Venous Catheterization, Central OR Central Catheterization OR Catheterization, Central OR Catheterizations, Central OR Central Catheterizations OR Central Venous Catheterization OR Catheterizations, Central Venous OR Venous Catheterizations, Central OR Central Venous Catheterizations OR Catheterization, Central Venous) [illegible]
GMT+0800 (N-Výh QÆeö•ô) hÀ}"~Ógœ : 5171

3: TS=(Intensive Care Units or Intensive Care Unit or Unit, Intensive Care or ICU Intensive Care Units) •ĐˆLeåg : Sun Jul 16 2023 18:27:29 GMT+0800 (N-V[illegible]

4: TS=Infections •ĐˆLeåg : Sun Jul 16 2023 18:28:37 GMT[illegible]

5: #2 OR #1 •ĐˆLeåg : Sun Jul 16 2023 18:29:08 GMT+080[illegible]

6: #5 AND #4 AND #3 •ĐˆLeåg : Sun Jul 16 2023 18:29:16

7: TS=(relative risk or cohort studies) •ĐˆLeåg : Sun Jul 1[illegible]
(N-Výh QÆeö•ô) hÀ}"~Ógœ : 951649

8: #6 AND #7 •ĐˆLeåg : Sun Jul 16 2023 18:30:56 GMT+08[illegible]

EMBASE:

Embase Session Results

No.	Query	Results
**#9**	**#7** AND **#8**	276
**#8**	**'relative risk'**:ab,ti OR **'cohort studies'**:ab,ti	141,565
**#7**	**#5** AND **#6**	19,403
**#6**	**#1** OR **#2** OR **#3** OR **#4**	41,553
**#5**	**'infections'**/exp	4,503,446
**#4**	**'catheterization, central venous'**:ab,ti OR **'venous catheterization, central'**:ab,ti OR **'central catheterization'**:ab,ti OR **'catheterization, central'**:ab,ti OR **'catheterizations, central'**:ab,ti OR **'central catheterizations'**:ab,ti OR **'central venous catheterizations'**:ab,ti OR **'catheterizations, central venous'**:ab,ti OR **'venous catheterizations, central'**:ab,ti	238
**#3**	**'central venous catheterization'**/exp	10,273
**#2**	**'catheter, central venous'**:ab,ti OR **'catheters, central venous'**:ab,ti OR **'venous catheter, central'**:ab,ti OR **'venous catheters, central'**:ab,ti OR **'central venous catheters'**:ab,ti	8,330
**#1**	**'central venous catheter'**/exp	32,291
