# Supplementary material for: Risk factors of central catheter bloodstream infections in intensive care units: A systematic review and meta-analysis
Source: PLoS One. 2024 Apr 23;19(4):e0296723. doi: 10.1371/journal.pone.0296723 (PMC11037535; doi:10.1371/journal.pone.0296723)
Supplement: S1 Table — (PDF) [file pone.0296723.s003.pdf]

Study quality of included studies based on the Newcastle-Ottawa

S1 Table. Study quality assessment of included case-control studies using NOS

Study	Selection				Comparability	Exposure			Score
	adequate definition of case	Representativeness of the cases	Selection of Controls	Definition of Controls		Ascertainment of exposure	Same method of ascertainment for cases and controls	Non- Response rate	
Li Liu 2022	★	★		★	★ ★	★	★		7
Kaichen Yan 2022	★	★		★	★ ★	★	★		7
Dongmei Hou 2021	★	★		★	★ ★	★	★		7
Hairong Yuan 2021	★	★		★	★ ★	★	★		7
Yuanye Li 2020	★	★			★ ★	★	★		6
Hongying Chen 2020	★	★			★ ★	★	★		6
Li, Qiao2020	★	★			★ ★	★	★		6
Huaming Peng 2020	★	★			★ ★	★	★		6
Hui-ying Yang 2018	★	★			★ ★	★	★		6
Xinqun Pan 2018	★	★			★ ★	★	★		6
Ye Liang 2018	★	★			★ ★	★	★		6
Shizhi Tian 2018	★	★			★ ★	★	★		6
Yanfang Yang 2017	★	★			★ ★	★	★		6
Zhen Tao 2017	★	★			★ ★	★	★		6
Shuiqin Cheng	★	★			★ ★	★	★		6

2016									
Bo Yang 2016	★	★			★ ★	★	★		6
Zhihua Jiang 2016	★	★			★ ★	★	★		6
Liyan Chen 2015	★	★			★ ★	★	★		6
Bing Liu 2015	★	★			★ ★	★	★		6
Baochun Zhou 2015	★	★			★ ★	★	★		6
Yinmei Liu 2014	★	★			★ ★	★	★		6
Xijiang Zhang 2012	★	★			★ ★	★	★		6
Li Xiao 2012	★	★			★ ★	★	★		6
Huihun Wang 2012	★	★			★ ★	★	★		6

Note: In the “Selection” and “Exposure” categories, a quality item of a study can be rated at most one “★”, and for the “Comparability” category, at most two “★”.
