# Supplementary material for: Risk factors of central catheter bloodstream infections in intensive care units: A systematic review and meta-analysis
Source: PLoS One. 2024 Apr 23;19(4):e0296723. doi: 10.1371/journal.pone.0296723 (PMC11037535; doi:10.1371/journal.pone.0296723)
Supplement: S2 Table — (PDF) [file pone.0296723.s004.pdf]

Study quality of included studies based on the Newcastle-Ottawa

S2 Table. Study quality assessment of included cohort studies using NOS

Study	Selection				Comparability	Exposure			Score
	Representativeness of the exposed cohort	Selection of the non-exposes cohort	Ascertainment of exposure	Demonstration that outcome of interest was not present at start of study		Assessment of outcome	Was follow-up long enough for outcomes to occur	Adequacy of follow up of cohorts	
Yiyue Zhong 2021	★	★	★	★	★ ★	★	★	★	9
Xiaoqing Shao 2018	★	★	★	★	★ ★	★	★	★	9
S.B.MISHRA 2017	★	★	★	★	★ ★	★	★	★	9
Zied Hajjej 2014	★	★	★	★	★ ★	★	★	★	9
Peng S 2013	★	★	★	★	★ ★	★	★	★	9
Hong Zhou 2012	★	★	★	★	★ ★	★	★	★	9
MatthewE. Lissauer 2012	★	★	★	★	★ ★	★	★		8
Daniela Bicudo 2011	★	★	★	★	★ ★	★	★		8

Note: In the “Selection” and “Exposure” categories, a quality item of a study can be rated at most one “★”, and for the “Comparability” category, at most two “★”.
